# Mechanisms of HTLV-1 persistence and transformation

**DOI:** 10.1038/sj.bjc.6605345

**Published:** 2009-09-29

**Authors:** M Boxus, L Willems

**Affiliations:** 1 National Fund for Scientific Research, Gembloux Agro-Bio Tech, Molecular and Cellular Biology, Gembloux, Belgium; 2 Interdisciplinary Cluster for Applied Genoproteomics (GIGA), University of Liège (ULg), Belgium

**Keywords:** HTLV-1, ATL, TAX, HBZ, transformation, immune escape

## Abstract

Adult T-cell leukaemia (ATL) is caused by the human T-cell lymphotropic virus type 1 (HTLV-1). HTLV-1 has elaborated strategies to persist and replicate in the presence of a strong immune response. In this review, we summarise these mechanisms and their contribution to T-cell transformation and ATL development.

Human T-lymphotropic virus type-1 (HTLV-1) belongs to the *Deltaretrovirus* genera of the Orthoretrovirinae subfamily. HTLV-1 is the first discovered human retrovirus, isolated in the early 1980s from peripheral blood samples of a patient with cutaneous T-cell lymphoma (Poiesz *et al*, 1980). Even if the exact number of sero-positive individuals is not known, an estimated 20 million people would be infected with HTLV-1 worldwide (de The and Kazanji, 1996). HTLV-1 is endemic in the Caribbean, Southern Japan, Africa, South America and Pacific islands. HTLV-1 is also present in Europe and North America where infection is epidemic. HTLV-1 is the etiological agent of an aggressive leukaemia, called adult T-cell leukaemia/lymphoma (ATL) as well as inflammatory disorders including tropical spastic paraparesis/HTLV-associated myelopathy (TSP/HAM), arthritis, uveitis, dermatitis, lymphadenitis and Sjögren's syndrome (Proietti *et al*, 2005). In addition to HTLV-1, other members of the *Deltatretovirus* genus are HTLV-2, -3 and -4 as well as bovine leukaemia virus (BLV). HTLV-2 was identified in cell lines derived from a patient with atypical hairy cell leukaemia although further studies failed to confirm the association of HTLV-2 with lymphoproliferative diseases. HTLV-3 and -4 subtypes have recently been identified in bushmeat hunters in Central Africa; infection by these two viruses being presently not associated to any pathology (Mahieux and Gessain, 2005). BLV is the causative agent of a B-cell neoplastic disease in cattle (reviewed by Gillet *et al*, 2007).

Most HTLV-1 carriers remain infected lifelong without developing any major clinical manifestation. After several decades, only a small proportion (2.1% for females and 6.6% for males) of HTLV-1-infected subjects will progress to ATL. The term ATL includes a spectrum of diseases that are referred to as smoldering, chronic, lymphoma and acute. ATL patients have atypical lymphoid cells with multilobulated nuclei (so-called flower cells) in their peripheral blood. ATL cells are consistently monoclonal with respect to proviral integration and originate from initial polyclonal/oligoclonal expansion of HTLV-1-infected cells. Leukaemia may progress from a smouldering phase to chronic and acute clinical manifestations. Acute and lymphoma subtypes show aggressive and rapidly fatal clinical courses with a median survival time of about 1 year. Although tumour cells are sensitive to conventional chemotherapy, patients rapidly relapse and become resistant to further treatment. Chronic and smouldering stages have a more indolent course and do not require chemotherapy (Proietti *et al*, 2005).

## HTLV-1-Encoded proteins

**Figure 1:**
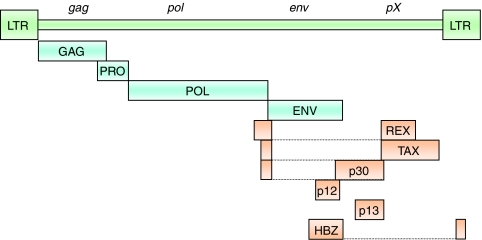
The HTLV-1 proviral genome.

Despite a relatively small genome (9 kb), HTLV-1 expresses multiple gene products by using both strands of its proviral genome and complex events of mRNA splicing (Figure 1). The HTLV-1 genome contains typical structural and enzymatic genes (*gag*, *pro*, *pol* and *env*) flanked by two long terminal repeats (LTRs). The long terminal repeats (LTR) are subdivided into three regions (i.e., U3, R and U5) that contain the *cis*-acting elements essential for viral gene expression: transcription factor-binding sites, transcription start and termination sites, polyadenylation and splicing sites. A region called pX, which is located between the *env* gene and the 3′-LTR, contains at least four partially overlapping reading frames (ORFs) encoding accessory proteins (p12^I^, p13^II^, p30^II^), the post-transcriptional regulator REX (ORF III) and the TAX transactivator (ORF IV). In addition, HBZ is encoded from the 3′ LTR in the complementary strand of the genome (Gaudray *et al*, 2002). Among all these regulatory proteins, TAX and HBZ proteins appear to have particularly important roles in viral persistence and pathogenesis, presumably through stimulation of continuous cell growth of infected cells in the presence of a strong immune surveillance.

### TAX

TAX (p40) activates viral transcription by interacting with triplicate enhancer elements (TxRE) located in the 5′-LTR. TAX is also a major factor mediating viral persistence and disease development. The oncogenic potential of TAX is supported by its ability to immortalise cells *in vitro*, stimulate colony formation in soft agar and produce tumours in transgenic mouse models. Within the cell, TAX exerts its functions by complex formation with more than a hundred cellular proteins (Boxus *et al*, 2008). We recently proposed that the intrinsically disordered structure of TAX allows a wide variety of conformational changes enabling binding diversity and recognition of differently shaped protein partners. Thereby, TAX triggers a plethora of cell-signalling pathways, reprograms the cell cycle, interferes with checkpoint control and inhibits DNA repair. The recent observation that TAX also modulates the miRNAs environment adds another level of complexity to its biological functions (Bouzar and Willems, 2008). Altogether, these pleiotropic activities of TAX cooperate to promote infected T-cell proliferation, generate DNA abnormalities and lead to subsequent transformation.

Among cellular pathways activated by TAX, CREB/ATF, NF-*κ*B and AP1 are thought to have predominant roles in T-cell proliferation and transformation. Molecular mechanisms sustaining these TAX activities have been reviewed recently (Boxus *et al*, 2008). Depending on the model, TAX mutants defective for the activation of these pathways are poorly oncogenic both *in vitro* and *in vivo*. For example, TAX-mediated NF-*κ*b activation stimulates expression of cytokines and their receptors such as interleukin 2 (IL2)/IL2 receptor (IL2R), IL9, IL13 and IL15/IL15R as well as members of the tumour necrosis factor receptor family. Thereby, TAX provides stimulatory signals resulting from the binding of ligands to their receptors and promotes cell proliferation as well as the initiation and maintenance of transformation (Grassmann *et al*, 2005; Sun and Yamaoka, 2005).

Besides stimulation of cell growth through signal transduction, TAX also directly subverts cell cycle progression by protein–protein interaction and transcriptional regulation of cell cycle-associated proteins. A major activity of TAX is stimulation of G1/S transition (Marriott and Semmes, 2005). Mechanistically, TAX increases the levels of type D cyclins in G1 and activates cyclin-dependent kinases (i.e., CDK4 and CDK6) through direct binding, leading to hyperphosphorylation of Rb, subsequent release of E2F transcription factor and accelerated transition from G1 to S. TAX also directly interacts with and promotes proteasomal degradation of Rb. Furthermore, TAX modulates expression of CDK inhibitors such as p18^INK4c^, p19^INK4d^, p21^WAF1^ and p27^KIP1^ and inactivates p15^INK4b^ and p16^INK4a^ through direct binding (Grassmann *et al*, 2005), thereby restraining their inhibitory activity toward CDKs.

Finally, owing to its PDZ-binding domain (PBM), TAX interacts with hDLG, which binds to the adenomatous polyposis complex (APC) tumour suppressor and enforces G0/G1 cell cycle arrest. To override regulatory functions of hDLG, TAX induces its hyperphosphorylation (Boxus *et al*, 2008). It is noteworthy that TAX mutants lacking the PBM are also impaired in immortalisation and transformation *in vitro* and *in vivo* (Xie *et al*, 2006).

### REX, p12, p13 and p30

REX (p27) is an RNA-binding post-transcriptional regulator that binds specifically to its *cis*-acting target sequence, the Rex response element (RRE), located at the 3′-end of sense viral mRNAs. REX promotes the export of the unspliced and singly spliced viral RNA species from the nucleus to the cytoplasm and inhibits splicing and transport of doubly spliced RNAs from the pX region (Kashanchi and Brady, 2005).

The four accessory proteins (p12^I^, p13^II^, p21^II^, p30^II^) are encoded by different pX mRNAs formed by alternative splicing events. p12 localises to the endoplasmic reticulum (ER) and regulates calcium-dependent signalling thereby activating nuclear factor of activated T-cell (NFAT) (Nicot *et al*, 2005). p12 also increases STAT5 transcriptional activity by interacting with the interleukin 2 receptor (IL2R). In addition, p12 interferes with MHC class I presentation by translocating newly synthesized MHC class I heavy chains towards the proteasome. An 8-kDa proteolytic cleavage form of p12 traffics to the cell surface, is recruited to the immunologic synapse following T-cell receptor (TCR) ligation, and downregulates TCR proximal signalling (Fukumoto *et al*, 2009). These multiple functions of p12 are thus likely to promote viral escape of immune surveillance as well as T-cell proliferation.

p30 is involved in the nuclear retention of the *tax/rex* mRNA leading to inhibition of virus expression and establishment of viral latency (Baydoun *et al*, 2008). In addition, p30 modulates expression of cellular genes involved in T-cell activation, apoptosis and cell cycle regulation through complex formation with p300/CBP and TIP60 (Nicot *et al*, 2005).

A truncated form of p30, p13 localises in mitochondria and alters membrane potential as well as reactive oxygen species (ROS) production (Silic-Benussi *et al*, 2009). On the other hand, p13 binds to farnesyl pyrophosphate synthase (FPPS), an enzyme required for the prenylation of Ras (Lefebvre *et al*, 2002).

### HBZ

The recently identified HBZ (HTLV-1 bZIP) factor acts as a negative regulator of TAX-mediated viral transactivation by heterodimerising with CREB, CREB2 and p300/CBP (Clerc *et al*, 2008). HBZ also modulates AP1 pathway by interacting with c-Jun, JunB and JunD. Similarly to TAX, HBZ interacts with proteasome subunits and may favour the delivery of cellular factors (e.g., c-Jun) towards the proteasome even in the absence of ubiquitination (Isono *et al*, 2008). Expression of the HBZ RNA leads to upregulation of E2F1 target genes and stimulates T-lymphocyte proliferation (Satou *et al*, 2006). Inversely, silencing of HBZ with shRNA decreases proliferation of HTLV-1-infected T cells and their ability to form solid tumours in mice (Arnold *et al*, 2008).

## Viral transmission and persistence

Although HTLV-1 infects many cell types *in vitro*, its preferential tropism *in vivo* includes CD4^+^ and CD8^+^ T-lymphocytes and dendritic cells. The ubiquitous glucose transporter 1 (GLUT1), neuropilin 1 (NRP1) and surface heparan sulfate proteoglycans (HSPGs) are cell surface receptors for HTLV-1 and are required for efficient entry (Takenouchi *et al*, 2007).

Transmission of HTLV-1 occurs through transfer of infected cells: from mother to child during breast-feeding, via sexual intercourse and through exposure to infected blood products or sharing of needles and syringes (Proietti *et al*, 2005). Cell-free HTLV-1 virions, which are not detected in the serum of infected subjects with currently available techniques, are indeed poorly infectious (Asquith and Bangham, 2008). Efficient transmission would thus rather rely on direct cell-to-cell contact, polarization of microtubule-organizing center (MTOC) which is triggered by TAX and formation of a virological synapse allowing the entry of viral proteins and genomic RNA into a new target T lymphocyte (Majorovits *et al*, 2008). Similarly to HIV, HTLV-1 also efficiently infects dendritic cells (DC) that subsequently transmit the virus to CD4^+^ T-cells in a biphasic manner (Jones *et al*, 2008). During the first day after viral exposure, DCs capture and transfer virions to CD4+ T cells (trans-infection). At later times, T cells are primarily infected by transmission of *de novo*-produced virus from infected DCs (*cis*-infection).

How HTLV-1 persists indefinitely in the infected hosts despite the presence of an active immune response is still intriguing. The current views about this apparent paradox postulates that virus rarely replicates via the infectious route but rather through mitosis of infected cells (Asquith and Bangham, 2008). As described in the previous paragraph, accessory and regulatory viral proteins are able to stimulate cell cycle progression thereby maintaining viral load in the host. Measurement of lymphocyte dynamics in HTLV-1-infected subjects shows that T-cell proliferation is increased compared with controls. This proliferative burst is correlated with the expression of viral proteins *ex vivo* (Asquith and Bangham, 2008). A recent study also reveals that the virus preferentially integrates into transcriptionally active genomic regions allowing favoured expression of viral proteins *in vivo* (Meekings *et al*, 2008).

Inoculation of HTLV-1 molecular clones in a rabbit model reveals that regulatory and accessory proteins (i.e., p12, p13 and p30) are required for efficient infectivity, viral spread and persistence *in vivo* (Matsuoka and Jeang, 2007). In contrast to TAX and HBZ, these accessory proteins, despite having an important role, are very poorly expressed in HTLV-1 infected cells isolated from patients. TAX has been dogmatically considered to be the main and only viral factor promoting persistence and pathogenesis but, recently, compelling evidences indicate that HBZ is clearly another key mediator. Out of the rabbit model, it appears that *tax/rex* and *gag/pol* mRNA levels peak early after infection and then progressively decrease. Conversely, *hbz* mRNA is detected later but increases over time. In primary T-cells isolated from asymptomatic carriers or ATL patients, *hbz* mRNA is highly expressed whereas *rex/tax* mRNAs are poorly, or not at all, detectable. Moreover, during ATL progression, the 5′LTR but not the 3′ LTR is frequently deleted or methylated, thereby overcoming TAX expression. Actually, TAX is a major target of cytotoxic T-cells and elicits a strong immune response that counterbalances infected cell propagation. In contrast, although HBZ is an immunogenic protein, HBZ-specific CTLs seem unable to efficiently eliminate HTLV-1-infected cells (Suemori *et al*, 2009).

A feedback loop coordinates TAX and HBZ expression: TAX activates HBZ, which, in turn, represses TAX-mediated transactivation. HBZ might thus prevent exaggerated TAX expression and allow infected cells to evade an immune response. Paradoxically, TAX expression is required to promote infected cell proliferation and viral persistence, through release of E2F factor and enforced G1/S transition. However, the ability of *hbz* mRNA to upregulate E2F1 gene transcription might supplant TAX-mediated release of E2F to promote continuous cell proliferation in latter steps of viral persistence (Satou *et al*, 2006).

Altogether, these data thus indicate that (1) HTLV-1 persistence relies on a subtle equilibrium regulated by the expression of viral proteins stimulating cell-growth in infected cells and their elimination by cytotoxic T-lymphocytes; (2) TAX and HBZ cooperate to promote infected cell proliferation in the early and late steps of infection and (3) silencing of viral expression allow escape from immune response.

## Role of genomic instabilities in transformation

Why only a small fraction of infected subjects will develop an ATL still remains an unanswered question. It is likely that continuous replication of HTLV-1 and promotion of cell proliferation ultimately leads to genomic instability and generates chromosomal abnormalities, a characteristic hallmark of ATL cells. Among regulatory proteins, TAX promotes infected T-cell transformation *in vitro* and *in vivo*. TAX has both clastogenic and aneuploidic effects that are thought to contribute to its oncogenic potential (Matsuoka and Jeang, 2007; Boxus *et al*, 2008).

Through its interaction with TAX1BP2 and RanBP1, TAX induces supernumerary centrosomes and causes multipolar mitosis, a common cause of aneuploidy. TAX also promotes unscheduled degradation of securin and cyclin B1 by interacting with CDC20-associated anaphase-promoting complex (APC). This process can lead to faulty chromosome segregation and ensuing aneuploidy. Mammalian cells have evolved the mitotic spindle assembly checkpoint (SAC) that is the guardian of genome euploidy. TAX binds to MAD1, an integral constituent of the SAC machinery, and impairs its activity. TAX thus promotes aneuploidy by interacting with proteins that monitor chromosomal segregation during mitosis (Matsuoka and Jeang, 2007).

How TAX induces clastogenic DNA damage is far from being well understood. On one hand, TAX represses expression of DNA polymerase-*β* that is involved in base excision repair and inhibits nucleotide excision repair induced by UV irradiation (Marriott and Semmes, 2005). On the other hand, TAX represses expression of the human telomerase reverse transcriptase (hTERT) and subverts Ku80 activity, thereby reducing protection from double-strand breaks (DSB) as well as telomere extension (Matsuoka and Jeang, 2007). This mechanism could explain why chromosome end-to-end fusions are frequently observed in HTLV-1-infected cells.

When cells undergo DNA damage, a complex network of signalling molecules, the DNA damage response (DDR), is activated. The DDR signalling network is often constitutively activated in premalignant lesions. This pathway is orchestrated by ATM, ATR and DNA-PK kinases whose substrates (e.g., CHK1, -2, p53, H2AX, 53BP1) delay cell cycle progression, promote DNA repair or even elicit permanent proliferative arrest (i.e., senescence) and apoptosis in case of extended, irreparable, DNA lesions. *De novo* TAX expression causes checkpoint activation and cell cycle arrest in G1 and G2/M phases of the cell cycle, consistent with the fact that TAX triggers genome instability (Marriott and Semmes, 2005). Our unpublished data indicate that TAX-dependent acceleration of the cell cycle induces replicative DNA damage resulting in constitutive DDR pathway activation in TAX-transformed cells as well as in T lymphocytes isolated from ATL patients (Boxus M and Willems L, unpublished data).

Growth arrest and apoptosis controlled by the DDR pathway may appear contradictory with the ability of the virus to sustain continuous cell proliferation. Nevertheless, permanent activation of DDR signalling creates a selection pressure that may be breached by cells deficient for ATM, CHK2, p53 or 53BP1 (Bartek *et al*, 2007). This mechanism, termed checkpoint adaptation, may allow HTLV-1-infected cells to proliferate with DNA damage, experience genetic instability and ultimately evolve to ATL clones. Although only a small percentage of ATL patients have genomic p53 mutations, the TAX protein interferes with the DDR pathway through inactivation of p53 (for a review see (Tabakin-Fix *et al*, 2006)). TAX also restricts activity of ATM, DNA–PK, CHK1 and CHK2-mediated checkpoints at G1 or G2/M (Marriott and Semmes, 2005; Chandhasin *et al*, 2008; Durkin *et al*, 2008). As a result, TAX limits cell cycle arrest induced by exogenous DNA-damaging agents. Therefore, it is anticipated that TAX counteracts the DDR pathway activation induced by its own DNA damage, resulting in checkpoint adaptation and fixation of genomic alteration. Such a model thus provides a rationale for genomic damage in ATL cells.

## Conclusions

**Figure 2:**
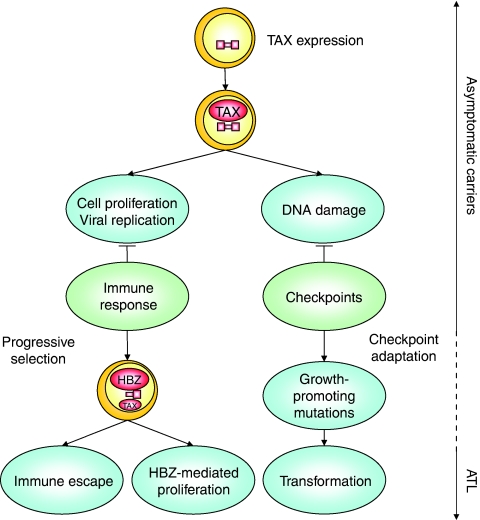
Role of TAX and HBZ in viral persistence and ATL development. In asymptomatic carriers, TAX stimulates viral and cellular gene expression, thereby promoting T-cell proliferation and allowing viral replication. The host immune response efficiently destroys most lymphocytes expressing viral antigens and selects for cells lacking or expressing low levels of TAX. Infected cell proliferation is then progressively promoted by HBZ, another viral factor encoded by the complementary strand. Forced and sustained cell replication by TAX generates DNA damage that activates checkpoints, a second barrier to transformation. In rare cases, this barrier may be breached through checkpoint adaptation mechanisms, allowing fixation of growth promoting mutations and subsequent ATL development.

HTLV-1 has developed sophisticated mechanisms to ensure persistence and, perhaps as a bystander effect, pathogenesis *in vivo* (Figure 2). As infection of new target cells is inefficient, viral replication occurs mainly through mitotic division of provirus carrying lymphocytes. HTLV-1 regulatory factors such as TAX and HBZ allow favoured proliferation of infected cells. HBZ, p12 and p30 either reduce viral expression or inhibit immune recognition. Permanent TAX-induced proliferation and abnormal expansion of infected cells generate DNA lesions characteristic of ATL. Inhibition of host checkpoint machinery allows further proliferation of infected cells harbouring DNA damage. Progressive stabilisation of these abnormalities provides an increased proliferative capacity to the infected cells and ultimately leads to ATL.
